# Regulatory Role of Redox Balance in Determination of Neural Precursor Cell Fate

**DOI:** 10.1155/2017/9209127

**Published:** 2017-07-18

**Authors:** Mohamed Ariff Iqbal, Eftekhar Eftekharpour

**Affiliations:** The Regenerative Medicine Program, Spinal Cord Research Centre, Department of Physiology and Pathophysiology, University of Manitoba, Winnipeg, MB, Canada R3E 0J9

## Abstract

In 1990s, reports of discovery of a small group of cells capable of proliferation and contribution to formation of new neurons in the central nervous system (CNS) reversed a century-old concept on lack of neurogenesis in the adult mammalian brain. These cells are found in all stages of human life and contribute to normal cellular turnover of the CNS. Therefore, the identity of regulating factors that affect their proliferation and differentiation is a highly noteworthy issue for basic scientists and their clinician counterparts for therapeutic purposes. The cues for such control are embedded in developmental and environmental signaling through a highly regulated tempo-spatial expression of specific transcription factors. Novel findings indicate the importance of reactive oxygen species (ROS) in the regulation of this signaling system. The elusive nature of ROS signaling in many vital processes from cell proliferation to cell death creates a complex literature in this field. Here, we discuss the emerging thoughts on the importance of redox regulation of proliferation and maintenance in mammalian neural stem and progenitor cells under physiological and pathological conditions. The current knowledge on ROS-mediated changes in redox-sensitive proteins that govern the molecular mechanisms in proliferation and differentiation of these cells is reviewed.

## 1. Introduction

The central nervous system (CNS) consists of the brain and spinal cord, which are comprised mainly of neurons, astrocytes, oligodendrocytes, and microglial cells. The earliest evidence that proliferating cells contribute to postnatal neurogenesis was proposed in mid-1960s [1, 2]; however, it took three more decades to find evidence of proliferating multipotential neural stem and progenitor cells in cell cultures of the embryonic and adult mammalian brain [3, 4] and spinal cord [5]. Shortly after these reports, other elegant studies showed the detailed anatomical location of these cells that are collectively known as neural precursor cells (NPCs) [6]. The NPCs play an important role in the generation of glial and neuronal cells in development and also function as a reservoir for normal tissue turnover [7]. The involvement of NPCs in memory formation and their capacity to proliferate and differentiate into different nervous tissue cells have inspired extensive research in the pursuit of an ultimate cure for the treatment of many diseases that are often associated with neural cell death, including neurodegenerative diseases, stroke, and spinal cord injury. Despite more than two decades of research, the details on factors that regulate NPC proliferation and differentiation are not quite clear. Neurotrauma and stroke have been shown to affect NPC population through a mixture of promoting cell proliferation and inhibitory factors for migration and appropriate differentiation [8, 9]. However, despite some increase in NPC proliferation in response to injury, the extent of their contribution towards efficient cell replacement and tissue repair remains very limited. Although the negative effects of trauma on NPCs have been attributed to the hostile posttrauma extracellular milieu [10, 11], the underlying mechanism involved in the postinjury rise in NPC proliferation remains unexplored. The identification of these factors that increase the capacity of NPCs for proliferation may lead to the identification of novel therapies.

Reactive oxygen species (ROS) are naturally generated in the mitochondria as an inevitable part of the oxidative phosphorylation respiration. The overall level of ROS is increased after any stress conditions including neurotrauma, creating a dual-edge sword that induce the removal of damaged tissue and initiate the repair process. ROS and their contribution to biological systems can be compared to the need for table salt in our diet; while a moderate amount of salt is needed, excessive quantity will undermine our health. In this manuscript, we aim to review the current literature on potential involvement of ROS in the regulation of NPC proliferation and differentiation. We will discuss some of the underlying signaling systems and antioxidant systems that have been shown to play a role in these processes.

## 2. Neural Stem and Progenitor Cells or Neural Precursor Cells (NPCs)

The NPCs are responsible for the normal turnover of the neural cell tissue. The common cardinal properties of the NPCs are their ability to self-renew and their capacity to differentiate to different neural cells. The formation of new neurons in adults is specifically localized to two regions: the subventricular zone (SVZ) and the subgranular zone (SGZ) in the dentate gyrus of the hippocampus in the CNS. The SVZ niche has been well studied in detail and is responsible for the generation of new neurons for the olfactory bulb. There are four cell types found in this region: the ependymal cells (Ep) line the ventricle and separate the cerebrospinal fluid from the SVZ. A highly proliferating cell type, known as neuroblasts or type A, is arranged in a chain [12]. These cells are migratory in nature and move in the rostral migratory stream to reach the olfactory bulb. A second type identified by their slow proliferation rate is known as type B cells. These cells are large quiescent cells with long processes and are located in close proximity next to the ependymal cells. These cells display a glial phenotype and contain GFAP intermediate filaments. Type B cells generate type C cells that are scattered in SVZ, representing transiently amplifying cells. Type C can divide once or twice and give rise to type A neuroblasts [13]. Thus, the SVZ is a region with a heterogeneous cell population, each with a different proliferative capacity. A recent study elegantly shows that a distinct subtype of NPCs in SVZ directly responds to signals originated from the hypothalamus ventromedial nucleus (satiety centre) [12]. Direct innervation of SVZ was shown to induce or impede cell proliferation in specific subtypes of NPCs mediated by the availability or the scarcity of food. The authors postulate that this may specifically affect the neuronal population of the olfactory bulb neurons for the animal [12]. Understanding the factors that regulate NSC proliferation can be used for the identification of novel therapies.

## 3. ROS and Oxidative Stress

Reactive oxygen species (ROS) are produced as a by-product of cellular metabolism. However, ROS production in aerobic organisms is majorly integrated as a signaling system rather than a negative regulator [14]. ROS levels are tightly regulated by a variety of means resulting in a balance between ROS production and their consumption known as redox balance. This balance is cell specific and reflects the cellular metabolism, specific function, and even the stage of cell cycle. For instance, young/proliferating cells have the highest levels of reducing antioxidants and therefore have a more reduced redox balance. As the cells proceed to differentiation, redox balance becomes more oxidized in aged/dying cells [15]. The redox balance also determines the cells' function, for example, immune cells that are involved in the phagocytosis of foreign pathogens generate high levels of ROS [16], while hepatocytes are equipped with reducing antioxidants to neutralize the many xenobiotics entering our body through the gastrointestinal system [17].

There are three major sources of ROS in the cells: mitochondria, cell membrane, and endoplasmic reticulum (ER). One of the most common forms of ROS, the highly reactive superoxide anion [O_2_^•^]^−^, is formed in mitochondrial complex III (cytochrome C oxidase) and complex I, located in the inner membrane of mitochondria as the components of mitochondrial electron transport chain [18, 19]. Additionally, cytosolic superoxide is produced by nicotinamide adenine dinucleotide phosphate (NADPH) oxidase (NOX) located in the cellular plasma membrane and the endoplasmic reticulum (ER) [18]. Superoxide anions can react with nitric oxide (NO) to generate the highly damaging peroxynitrite (ONOO^−^) [20]. Superoxide is enzymatically converted to hydrogen peroxide (H_2_O_2_) by superoxide dismutase located in the cytosol (SOD1) and mitochondria (SOD2). Despite lower reactivity of H_2_O_2_, this ROS has a longer half-life and can diffuse across biological membranes [21]. These properties make H_2_O_2_ an important player in cellular signaling. The damaging effects of H_2_O_2_ resulted from its conversion to hydroxyl radical (OH^•^) in the Fenton reaction catalyzed by Ferrous ion (Fe_2_^+^) [22]. The hydroxyl radical (OH^•^) is the most reactive and damaging free radical in biological systems and can extract electrons from other molecules, including membrane phospholipids. This results in the formation of lipid radicals that generate a chain reaction of lipid radical and lipid peroxide formation. Although vitamin E has been credited for scavenging lipid free radicals, the vitamin E itself is converted to *α*-tocopherol radical that must be quenched by vitamin C and thiol antioxidants [23]. Lipid peroxides can also go under the Fenton reaction and produce more hydroxyl radicals that exacerbate the cellular damage [24]. Therefore, the regulation of the peroxide (H_2_O_2_ and ROOH) level is a critically important task in maintaining cellular health. Thiols containing peptides and proteins are the prime antioxidant pool involved in the management of peroxides. In the following sections, we will examine the known effect of ROS in cell signaling, especially in the context of NPC cell proliferation, and will review some of the antioxidants that have been shown to affect NPC proliferation and differentiation.

## 4. Role of ROS in Cell Signaling: A Basic Understanding

Although the ROS-mediated damage has been well studied, developments of new tools and techniques during the last two decades have increased our knowledge of the ROS physiological importance in the cells. In prokaryotes, several transcription factors are directly regulated by ROS-mediated oxidation of their thiol groups located on cysteine residues [25]. The same principle applies in higher organisms where the distribution of cysteine is disproportionally high. The human proteome encodes about 214000 cysteine residues, reflecting a selective enriching of this amino acid during evolution [26]. The importance of cysteine in cell physiology has been related to the chemical property of sulfur that enables stable covalent bonds with major elements (C, N, O, H, and P) and transitional metal ions [27]. The thiol group in cysteine is readily oxidized by reactive ROS such as H_2_O_2_ and forms sulfenic acid (−SOH). This is highly unstable and is quickly converted to a disulfide bond. This step is reversible by thiol antioxidants; however, under excessive oxidative conditions, cysteine is further oxidized and forms the irreversible sulfinic (−SO2H) or sulfonic (−SO3H) acid [28]. Nitrosylation and glutathionylation are other forms of oxidative modification of cysteine residues that lead to changes in the structure and function of the cellular proteins [29]. Methionine is another thiol containing amino acid that responds to changes in the redox state of the cell by transient oxidation (methionine sulfoxide) and reduction by methionine oxide reductase [30]. These properties offer a unique position for cysteine/methionine as the interface between the extracellular environment (exposome) and the cellular proteome mediated through ROS. This is well exemplified in the original discovery of NPCs by their responsiveness to growth factors such as epidermal growth factor (EGF) and basic fibroblast growth factor (bFGF2) [31]. The effect of these growth factors is mediated through activation of NOX and generation of superoxide radical. This is quickly converted to H_2_O_2_ that will then activate the downstream signaling by *activation* of tyrosine kinases and concurrent *inactivation* of phosphatases to optimize the effect of the extracellular factors [32]. Our lab has recently shown that the level of ROS can affect cell-signaling machinery between cell survival and cell death [33]; therefore, the levels of ROS must be tightly regulated. We further showed that oxidative modification of methionine residues in cathepsin L may be responsible for a balance between the protective autophagy and apoptosis [34]. The thiol group in glutathione, thioredoxin, and glutaredoxins has a low redox potential enabling them to act as an electron buffering pool and therefore a central player in the regulation of cell signaling. The redox sensitivity of signaling proteins in cell proliferation has not been adequately investigated. New advances in the field of redox biology will identify new players in this field.

## 5. ROS as a Mediator in Growth Factor Response and Cell Proliferation

Several extracellular signals such as growth factors trigger cell proliferation. Extracellular ligand binding activates receptor tyrosine kinases (TrK), and sequential phosphorylation of its components relays the stimulus intracellularly to initiate the cell cycle machinery. Phosphatases act on kinases to prevent continuous stimulation and provide temporal and spatial signal specificity. Although the profile of underlying proteins involved in this process are well identified, recent advances in redox biology indicate the key role of ROS in the feed-forward amplification of the signaling cascade [35]. The identification of cysteine residues in the active site of these key signaling proteins mediate the redox regulatory of ROS and thiol antioxidant systems in these processes. Growth-promoting external stimuli that can elevate intracellular ROS levels predominantly originate from plasma membrane-bound NADPH oxidase (NOX). These ROS signals result in the inhibition of protein tyrosine phosphatases (PTP) in their vicinity, through oxidation-driven structural changes. This inhibition is reversible with reducing agents or proteins. PTPs at these sites are required to modulate the receptors' sensitivity for growth factors. This phenomenon is involved in the regulation of cell proliferation and is affected by cellular seeding density in cell culture conditions. At low density, elevated ROS levels in growing cells inhibit PTP activity [36]. Thus, TrK function is promoted/prolonged in these conditions, resulting in increasing cell proliferation rate. Cell-cell contact is known to decrease cell proliferation rate through ROS signaling modulating receptor phosphorylation/dephosphorylation activity. Such “contact inhibition” is associated with decreased intracellular ROS levels which activate phosphatases to ameliorate growth factor signaling in the cell culture system [37, 38]. Growth-promoting external stimuli can elevate intracellular ROS levels, through NOX in the plasma membrane.

EGF receptor is one of the well-studied signaling pathways that is involved in the regulation of NPC proliferation in developing mouse brain [31]. The activation of EGF receptors and the enhancement of cell proliferation in this system is known to be mediated by transient increase in H_2_O_2_ levels via small GTPase Rac1-stimulated NOX1 [39]. H_2_O_2_ elevation is crucial to relay the changes in the phosphorylation status of tyrosine receptor proteins (EGF receptor and phospholipase C-*γ*1) for the activation of intracellular signaling pathways. Notably, the inhibition of H_2_O_2_ production by increased catalase activity decreased the proliferation of human epidermoid carcinoma cells [40]. Besides EGF, the presence of bFGF receptors on NPCs is associated with the “stemness” of these cells resulting in colonial expansion in vitro in response to these growth factors [31]. Platelet-derived growth factor (PDGF) receptor (PDGFR) also plays a role in the development of CNS and neuroprotection [41]. PDGFR is also known to regulate oligodendrocyte progenitor formation and neuronal specification [42, 43]. Naïve NPCs do not express PDGFR but the expression of this receptor is rapidly increased in a subset of NPCs that are fated towards immature neurons and oligodendrocytes. PDGF also stimulates neuronal fate adaptation in dividing neural progenitor cells by promoting the expansion of immature neurons [44]. The mitogenic effect of PDGFR is similar to the activation of EGFR through transient elevation of intracellular H_2_O_2_ levels by NOX1. NOX1-mediated H_2_O_2_ elevation is also associated with induced proliferation in rat smooth muscle cells and human hepatic stellate cells [45, 46]. A direct relevance of nontoxic H_2_O_2_ addition (2–4 *μ*M) and high proliferation of NPCs has been reported where inhibition of NOX decreases NPC proliferation and neurogenesis [47]. Conversely, a previous report claims that H_2_O_2_ treatment decreases NPC proliferation [48]; however, it is noteworthy that concentrations of H_2_O_2_ used in this study were beyond its physiological levels (up to 100 *μ*M). Further confirmation of ROS inhibitory effect on NPC proliferation was shown after the upregulation of cellular antioxidant levels by N-acetylcysteine (NAC, a precursor of glutathione). Enhanced cellular reducing capacity increased O2A progenitor population (oligodendrocyte and type 2 astrocyte bipotent progenitors) and decreased oligodendrocyte differentiation [49], indicating that low ROS levels are important for cell proliferation and limit differentiation. Conversely, increase in mitochondrial-generated superoxide load or knockout of SOD2 was associated with decreased NPCs in developing mice brain [19]. Similarly, NPC-specific ataxia-telangiectasia mutated (ATM) knockout shows elevated intracellular ROS levels which negatively affected cell proliferation and neurogenesis [48]. This contradictory findings may indicate the delicate role of ROS balance in NPC expansion (proliferation) and differentiation. NPCs can be quiescent but will undergo activation with spiking ROS levels and proliferate to give rise to more NPCs. Similarly, differentiating cells have more ROS than its ancestor progenitors due to metabolic shift from aerobic glycolysis to oxidative phosphorylation [50, 51]. An in vitro report showed that post mitotic exit of neuroblasts to form immature neurons was associated with increased ROS levels and enhanced levels of mitochondrial electron transport proteins [52]. These information cumulatively indicate that ROS levels in a concentration-dependent manner modulate NPC proliferation and differentiation; however, the fine-tuning of signaling is regulated by many transcription factors.

## 6. ROS and NPC Proliferation

It is a well-accepted fact that all stem cells reside in areas with much lower oxygen than with the ambient oxygen levels, yet much of our knowledge on NPCs' biology comes from culturing these cells in normal tissue culture incubators containing the atmospheric 21% oxygen. The beneficial effect of lowered oxygen on NPCs was reported originally by Studer et al. [53], which showed increased proliferation and cell survival in these cells. Moreover, the positive effect of normal oxygen levels on the proliferation of NPCs has been extensively reviewed [54]. Additionally, cell differentiation was also affected by increased expression of neuronal markers when NPCs from developing midbrain were exposed to low oxygen pressure (3 ± 2%) [53]. The beneficial role of this physiological normoxia or “physoxia” has been partially credited to lower ROS levels in these conditions which improved cell survival of primitive neural stem cells [55]. The underlying mechanism in these conditions is well illustrated by positive regulatory domain-containing protein 16 (*Prdm16)*, which is preferentially expressed in primitive stem cell pools. A decrease in Prdm16 level increases ROS levels, causing depletion of hematopoietic and neural stem cells by increasing oxidative stress-mediated cell death. The regulatory role of Prdm16 is applied through its promoter control for hepatocyte growth factor (Hgf) gene with antioxidative properties. Thus, ROS elevation is correlated with decreased Prdm16 and downregulation of its downstream target Hgf [56]. Opposing observations of ROS-positive effects on NPC proliferation came from an elegant study by Le Belle's group [47]. In this report, the authors convincingly showed that highly proliferative neural stem cells contain increased levels of ROS, and experimental manipulation of their levels severely affected normal NPCs in vitro and in vivo [47]. However, these experiments were all conducted under normal atmospheric oxygen concentrations, posing the question whether these findings are relevant to physiological oxygen levels in the CNS.

The complexity of ROS involvement in proliferation and differentiation is further elevated by the type of ROS or the antioxidants used in these experimental conditions. An example of the different ROS-subtype effects on cell proliferation has been shown previously [57]. This group showed that in vascular smooth muscle cells, superoxide anions generated by xanthione/xanthione oxidase increased cell proliferation through the induction of Id3, a DNA-binding inhibitor protein. However, the cell proliferation was inhibited when cells are treated with H_2_O_2_, where the transcription factor gut-enriched Kruppel-like factor (GKLF) is induced. The group showed that Id3 : GKLF ratio regulates the cell proliferation, as overexpression of Id3 overcomes the inhibitory effect of GKLF. This study showed that specificity of ROS effects may be mediated through different redox-sensitive proteins.

ROS also are involved in NPC differentiation as was recently reviewed [51]. Overall, NPCs' proliferation has been inversely related to oxygen levels, and therefore in hypoxic zones, NPCs remain mostly proliferative. Upon the induction of mitochondrial activity and oxidative phosphorylation, the rise in ROS levels or exposure to stressful conditions inhibits proliferation and promotes cell differentiation [51]. Understanding ROS production and regulation in NPCs provide a window of opportunity to optimize cell proliferation and differentiation.

## 7. ROS Regulated Proteins and Transcription Factors

Redox signaling is a fast-paced and transient process in which electrons are transferred between the oxidizing ROS and redox-sensitive proteins, such as tyrosine kinases and phosphatases [58]. The interaction between ROS and these signaling molecules results in the transduction of external signals to NPC proliferation and differentiation. A sophisticated antioxidant system is required to maintain the balance between ROS levels and available antioxidants. Several transcription factors are known to be activated by the redox status of the cell. These include ATM, FoxOs, Nrf2, HIF1*α*, and APE1 that are known to regulate redox-driven signals with regard to NPC fate determination.

### 7.1. ATM Regulates NPC Self-Renewal and Differentiation

Ataxia-telangiectasia mutated (ATM) is a serine/threonine protein kinase involved in redox balance, DNA repair, and cell proliferation [59]. ATM was reported to be essential for adult neurogenesis as high levels of ATM expression are seen in neuronal progenitors, which decreases during differentiation, suggesting a role for ATM in the maintenance of proliferating NPCs. Ataxia-telangiectasia patients exhibit an aberrant neuronal differentiation, which is possibly due to excessive yet abnormal proliferation of neuronal progenitors [60]. Under normal condition, elevated ROS levels activate ATM, which leads to the downstream activation of p53, causing senescence in proliferating cells [61]. ATM regulates ROS levels in self-renewing NPCs, as its knockout increases ROS levels leading to oxidative stress, resulting in decreased proliferation of NPCs which can be rescued by antioxidants or p38 MAPK inhibitor [48]. This study suggests that excessive upregulation of ROS levels is detrimental for cell proliferation.

### 7.2. FoxOs

FoxO proteins (forkhead transcription family O) are a group of transcription factors that have consensus binding site. FoxO is known to double life expectancy in *Caenorhabditis elegans.* Interestingly, there is a strong association with human longevity and the involvement of a genetic variant of FoxO3A. Animal studies suggest a correlation between FoxO3 and insulin and insulin-like growth factor signaling [62, 63]. FoxO1 is essential in the activation of redox-sensitive Oct4, a key modulator of pluripotency in stem cells, thus maintaining their embryonic pluripotency [64]. There is a cell-specific regulation of FoxO in NPCs where FoxO1 is exclusively expressed in slow-proliferating NPCs (type B) and is excluded in doublecortin positive (Dcx+) neuronal progenitors (type A); this indicates a cell stage-specific expression/regulation [65]. Thus, FoxO1 is associated with the repression of differentiation and the maintenance of stemness and regulates the stem cell reservoir. FoxO3 is also known to maintain NPC pool homeostasis by regulating proliferation and differentiation [66]. An evolutionarily conserved interaction of MST1-FOXO plays an important role in oxidative stress response in mammalian neurons. A similar interaction of orthologs CST1-DAF-16 is reported to increase life span in the nematode worm [67]. The oxidative stress response is mediated through FoxOs in many stem cell types, including hemopoietic stem cells [68]. A direct deletion of FoxOs in neural precursor cells results in megalocephaly or enlarged brain, suggesting increased proliferation of NPCs that can result in the depletion of NPC pool in the adult brain [69]. FoxO-null mice display an increased level of ROS in their NPC pool which results in reduced self-renewal of the stem cell supply [69]. Interestingly, FoxOs are induced in neurodegenerative diseases and spinal cord injury, which can potentially promote recovery [70, 71].

Cumulatively, the current literature indicates a regulatory role for FoxOs on NPC proliferation and differentiation. This includes the regulation of antioxidant systems [72], which control ROS levels that are ultimately involved in maintaining the NPCs' reservoir as well as their fate determination. Understanding such complexity can have a potential application in aging and neurotrauma.

### 7.3. HIF1*α*

Hypoxia is a condition when a tissue receives less oxygen than its surrounding region. The occurrence of hypoxia is physiological during development and pathological in neurotrauma. During embryonic development, most of stem cell niches experience hypoxia [73] that requires adaptive changes to survive these conditions. Hypoxia inducible factor-1 alpha (HIF1*α*) is an oxygen sensor that is induced by low oxygen levels but is degraded rapidly in normoxia [74]. Hypoxia can increase NPC proliferation and reduce apoptosis by HIF1*α* dependent and independent pathways [55]. HIF1*α* is essential in maintaining neural stem cells in mice adult SVZ and is required for maintaining the vasculature by inducing vascular endothelial growth factor (VEGF) [75]. The role of HIF1*α* in CNS development is well documented by its involvement in vasculogenesis as well as embryogenesis [76]. Under pathological conditions such as stroke, ROS are generated after the interruption of blood supply (hypoxia) and again after the restoration of blood supply (reperfusion). Although the damaging effects of ROS on cellular health has been well documented [77], interestingly, ROS generation has been also shown to be correlated with increased neurogenesis [78–80]. HIF1*α* is also known to maintain the NPCs in a quiescent state in the adult and embryonic brain [75, 81]. ROS induces transactivation of NF*κ*B and in turn induces HIF1*α* promoter in pulmonary artery smooth muscles [82]. Redox regulation of HIF1*α* is well established through regulation of its stability by ROS levels [83, 84]. Therefore, oxidative modification of this master transcription factor can affect the downstream genes including VEGF, p21, p53, and Bcl-2. As a therapeutic approach, transplanting HIF1*α* overexpressing NPCs improved neurological function in rat after cerebral ischemia via increasing survival and providing microvascularization [85]. Thus, hypoxia and ROS levels can play a crucial role in defining stem cell properties and increasing cell proliferation, which can be utilized for novel therapeutic strategies.

### 7.4. PTEN-PINK1

Phosphatase and tensin homolog (PTEN) is a tumor suppressor gene, which is frequently mutated in many human cancers [86, 87], but also has been associated with social deficits and autism spectrum disorder in mice [88]. The presence of PTEN results in NPCs exiting cell cycle at the G_0_ phase and increased self-renewal by AKT1 downregulation [89]. PTEN deficiency causes hyperproliferation but does not cause stem cell depletion. The PTEN depletion causes cell proliferation irrespective of growth factor dependency [90]. PTEN is a redox-sensitive protein, which controls NPC proliferation. Growth factors such as EGF and PDGF stimuli can cause elevated ROS mediated by NOX which reversibly oxidize PTEN to promote cell proliferation [91]. This reversible oxidative inhibition of PTEN modulates the proliferation of NPCs when required. This phenomenon has been taken advantage of by several cancer cells. As discussed before, Nox2-associated growth factor signaling for proliferation occurs via PTEN inhibition (transient oxidation). A study reported that adult hippocampal progenitors are maintained by oxidizing PTEN upon FGF signaling [92]. Interestingly, PTEN knockout neurospheres show increased proliferation under basal conditions and do not further increase proliferation when exposed to elevated ROS conditions. In contrast, neurospheres from wild-type and PTEN heterozygous mice responded to H_2_O_2_ by increased hyperproliferation [47]. Thus, PTEN is involved in the generation of cancer-initiating stem cells by adaptation of reduced growth factor responsiveness. PTEN-induced putative kinase 1 (PINK1) is a downstream of PTEN and is known to be involved in mitochondrial function. PINK1 knockout results in increased ROS generation and thus oxidative stress [93, 94]. In a recent study, PINK1 is shown to increase during normal mice development and its deletion results in decreased gliogenesis, without affecting NPC proliferation and neuronal or oligodendrocyte differentiation. Given the fact that astrocyte differentiation pathway is altered but NPC proliferation is unaffected upon PINK1 knockout, understanding the underlying mechanisms that regulate the ROS-PINK1protein control of gliogenesis remains to be explored. Mitochondrial ROS generation was not changed during in vitro NPC differentiation [95]. This is not the case in mouse embryonic fibroblasts, where mitochondrial ROS levels and oxidative stress are exacerbated upon PINK1 global knockout [93]. The difference in mechanisms for such phenotype is yet to be explored. Overall, understanding PINK1 may reveal the mechanisms behind reactive astrogliosis.

### 7.5. p53

p53 protein functions as a tumor suppressor, and a negative controller of cell proliferation is mediated by p21, a key negative regulator of cell cycle and apoptosis in adult NPCs [96]. Although the role of p53 in the differentiation of embryonic stem cells has been shown [97, 98], its involvement in NPC differentiation remains to be identified. Conversely, a paralog of p53 known as p73 is crucial in maintaining the neurogenic pool by regulation of the NPCs' self-renewal and proliferation in both embryonic stage and throughout the adulthood [99]. A direct correlation of p53 in ROS generation in mouse embryonic NPCs has been reported [100]. The study suggests that a fine-tuning of ROS generation is controlled by p53. The study further reveals that elevated ROS is correlated with early neurogenesis and propagation of DCX+ precursor cells. Their data suggests that knockout of p53 is associated with increased ROS that in turn activates AKT-PI3Kinase pathway which forces the NPCs to commit to neuronal fate [100]. Although these results contradict the theory which says increased endogenous ROS is required for multipotent stem cell proliferation [47], the difference could come from the additional stress levels after p53 knockout that can cause enhanced oxidative stress beyond the threshold for proliferation and therefore affects the NPCs' fate commitment. P53 also regulates apoptosis as well as ROS regulation and NPC metabolism which eventually regulate proliferation.

### 7.6. Nrf2

Nuclear factor erythroid 2-related factor 2 (Nrf2) is a master transcription factor that induces a battery of genes with a common conserved sequence known as the antioxidant response element (ARE) in their promoters [101]. Nrf2 is dynamically regulated by ROS levels in neural stem cells [102]. Under normal conditions, Nrf-2 is quickly ubiquitinated and destined for degradation; however, when exposed to increased ROS levels during oxidative stress conditions, Nrf2 is translocated into the nucleus where it binds to ARE and increases a set of antioxidant proteins [103], including members of glutathione and thioredoxin systems. Quinone compounds such as tertiary butyl hydroquinone (tBHQ) can also increase Nrf2 protein stability and thus increase cellular defense [104]. The upregulation of Nrf2 is known to enhance NPCs' survival against any oxidative stress [104]. This has been shown in SVZ-derived NPCs in culture or after ischemia, which correlates with induced neurogenesis [103]. The effect of Nrf2 is partially mediated by Notch1 signaling, which is conserved in neurogenesis but not in gliogenesis [105]. A recent report suggests a putative role of Nrf2 in neuronal fate commitment through ROS signaling [106]. The group elegantly showed that physiological levels of ROS mediate Nrf2 concerted signaling that eventually affects NPCs' self-renewal and fate determination [106]. The exact role of Nrf2 on NPCs in neurodegenerative diseases for its potential therapeutic application remains to be investigated.

## 8. NPC Metabolism and Mitochondria

Cellular metabolism also affects NPC proliferation and differentiation. The early embryonic NPCs, also known as primitive NPCs, are mostly dependent on glycolysis [107]. As development progresses and nutritional requirements change, there is a shift from glycolysis to oxidative phosphorylation [108, 109]. Quiescent stem cells residing in the SVZ are similar to a primitive cell type and depend on glycolysis. Oxidative phosphorylation is the main form of metabolism in proliferating NPCs. However, a forced disruption of mitochondrial metabolism in NPCs will switch their metabolism back to glycolysis, resulting in their quiescence via p53 inactivation [110]. Increased oxidative damage in mitochondria that leads to mitochondrial DNA damage decreases self-renewal in NPCs, which can be rescued by the antioxidant N-acetyl cysteine (NAC) [111]. Another study by the same group in induced pluripotent stem cells provided similar observations including decreased proliferation with mitochondrial mutagenesis, an effect which could be rescued with mild concentration of NAC and mitochondria-targeted antioxidant mitoQ. Interestingly, higher levels of antioxidants resulted in decreased proliferation [112]. These studies suggest that high levels of ROS are detrimental for NPC proliferation and differentiation. This may also imply the potential application of antioxidants for diseases that are associated with abnormal changes in NPC proliferation [112]. Mitochondria are one of the major sources of cellular ROS, and certain ROS can leak through the membrane affecting the overall cell health. Mitochondrial structural dynamics are also known to affect ROS generation that can influence the self-renewal and differentiation in NPCs [106]. Sox2-positive NPCs contain elongated mitochondria and show lower ROS levels compared to Sox2-negative progenitors with fragmented mitochondria and higher ROS levels. Moreover, low ROS in Sox2+ NPCs contain reduced Nrf2 which enhances Notch signaling, and thus, NPCs self-renewal is maintained; whereas in Sox2-negative progenitors, ROS elevation activates Nrf2 signaling which results in reduced self-renewal and induced differentiation. Antioxidant NAC reversed the condition and increased cell proliferation [106]. A more recent study reveals ultrastructural evidence for mitochondrial function alterations in the adult NPCs. Rounded mitochondria display with rudiment OxPhos and electron transport chain (ETC) machineries in NPCs while intermediate progenitors (IPCs) have elongated mitochondria with functional OxPhos and ETC components [113]. This shows the metabolic shift in quiescent NPCs to become actively dividing IPCs. We could appreciate the effect of metabolism in altering ROS levels and thus fate determination.

## 9. ROS, Autophagy, and NPCs

Autophagy is a dynamic process regulating energy utilization and recycling damaged proteins and organelles. It is said that autophagy is a response to starvation condition, but a basal level of autophagy occurs naturally in all the cells. There is a developing interest to understand autophagy mechanism in NPCs in self-renewal and differentiation. Basal autophagy regulates mitophagy to keep the mitochondrial ROS production at bay [114, 115]. This is an essential step to maintain stem cell properties. Mammalian target of rapamycin (mTOR) signaling and other autophagy inducers also are known to regulate NPC maintenance [116, 117]. Redox control of autophagy in stem cells in physiology and pathophysiology are an emerging topic [118, 119]. FIP200 is an essential protein that initiates autophagy in NPCs. Specific deletion of FIP200 in NPCs results in defected SVZ and dentate gyrus in adult mice. Such deletion of FIP200 also results in the accumulation of p62, increased mitochondria, and elevated ROS levels. The impairment in self-renewal was rescued by p53 ablation or NAC treatment; however, the defected neurogenesis was not rescued by p53 ablation but was amendable to NAC [120]. This suggests the importance of autophagy in regulating ROS levels by controlling mitochondria in postnatal cells but not in embryonic NPCs. This may represent the differential mechanisms in different developmental stages. In a follow-up study in the same direction, the group tested few other autophagy regulators and found that Atg5 and Atg16L1 conditional deletion impaired autophagy but not p62 accumulation in postnatal NPCs. The accumulation of p62 in FIP200 null mice causes aberrant NPC properties and increased O_2_^•−^ levels by SOD1 inhibition. FIP200 and p62 double knockout resulted in the recovery of NPC properties and reduced ROS levels but no reduction in elevated mitochondria number [121]. This accumulating evidence shows how aberrant ROS production due to autophagy defect leads to decreased NPC health. This is relevant in any CNS diseases where autophagy is affected.

## 10. Regulation of ROS Levels: Involvement of Thioredoxin Family of Proteins

Protein oxidation-reduction is majorly governed by thiol-based protein systems such as thioredoxin and glutathione systems, and therefore, many potent antioxidants mimicking these thiols are used as potential therapeutic molecules. Thioredoxin family of proteins consists of reduced (active) thioredoxin (Trx), which physically interacts with peroxides or other substrate proteins and reduces their oxidized thiol groups. Two thiol groups in Trx active site (Cys32-Gly-Pro-Cys35) provide the reducing electrons for the reduction of oxidized proteins or the oxidizing ROS [122]. Trx is oxidized in these reactions but will be regenerated by Trx reductase using electrons from nicotinamide adenine dinucleotide phosphate (NADPH) [123]. Thioredoxin-interacting protein (Txnip) is a natural inhibitor, physically interacting with Trx1 and Trx2, preventing their physical interaction with the substrate [29]. Trx1-TrxR1 oxidoreductase pair is found in cytosol, but Trx2 and TrxR2 are specific to mitochondria with a similar function to their cytoplasmic counterparts [123]. Trx1 plays a crucial role in cell proliferation by reducing a key enzyme, ribonucleotide reductase, in DNA synthesis [124]; however, other reducing factors such as glutathione and dithiothreitol failed to replicate Trx effect on cell proliferation [125]. This indicates that Trx growth-promoting role may be mediated by other properties such as the regulation of cellular response to growth factors [126]. Trx is also involved in many cellular activities which is mediated by its reducing capacity for DNA binding in some transcription factors, as shown for Oct4 that controls cell proliferation [127]. Higher levels of Trx in some cancers have been linked to enhanced cell proliferation and resistance to oxidative stress, and therefore, Trx and TrxR are targeted in anticancer therapies [128, 129]. Trx is secreted from some cells through a leaderless pathway although the mechanism of action in extracellular space is not identified [130]. Supplementing the growth medium with Trx1 has been shown to increase cell proliferation in a series of solid tumors [126]. Yet there are currently no receptors identified for Trx; however, minimal uptake of Trx has been reported [126]. In the nervous system, Trx1 is secreted from astrocyte that has been linked to neuroprotection [131]. A recent study shows that Trx1 addition in vitro and in vivo increases NPC proliferation and promotes neurogenesis [132]. The study showed that the number of Dcx^+^ cells was increased in this study in embryonic NPCs [132]. New emerging evidences indicate that Trx effect on hematopoietic stem cell proliferation might be mediated through the regulation of ROS. A growing list of substrates for Trx gives an insight and depth of Trx influence in cell proliferation. For instance, its interaction with senescence-associated proteins ASK1 and Txnip prevents apoptosis induction and cell cycle arrest, respectively [133, 134]. The fact that Trx is stimulated by the antioxidant and oxidant response elements in its promotor region makes Trx a first response protein in stressful conditions [135]. The negative regulator of Trx, Txnip, has been correlated with the induction of oxidative stress through ROS production [136]. Oxidative stress or increased ROS can lead to Trx-Txnip dissociation, which can stabilize Txnip function in senescence as well as inflammasome activation [29]. Txnip has also been linked to reactive astrogliosis in diabetic retinopathy [137]. Thus, major antioxidant systems may contribute to therapeutic applications by modulating cell proliferation and differentiation.

## 11. Neural Stem Cells in Pathology

Acute neurotrauma, chronic neurodegenerative diseases, and aging are shown to affect NPC population [138] including increased proliferation in acute stroke and spinal cord injury [79, 139]. Conversely, depletion of NPCs or lack of their proliferation is a characteristic feature of chronic diseases such as Alzheimer's and Parkinson's diseases [140]. All of these conditions are associated with aberrant ROS signaling. Mild oxidative challenge by buthionine sulfoximine (BSO) and other oxidants has been shown to decrease self-renewal (proliferation) of NPCs and modulates multipotentiality by increasing astrocyte differentiation while decreasing neurogenesis. Increased Sirt1 is one of the factors for this effect at least in defected neurogenesis. Sirt1, a multifaceted nicotinamide adenine dinucleotide- (NAD-) dependent histone deacetylase, is involved in energy metabolism and transcriptional regulation [141]. Increased Sirt1 expression decreases Mash1 expression in an epigenetic manner [50]. In another study with similar concept, H_2_O_2_ treatment promoted cell death and inhibited the protective autophagy in Sirt1 knockout cells in mouse embryonic stem cells [142]. Such experimental conditions may be useful in mimicking the CNS pathological conditions with elevated oxidative stress. Aging results in reduced stem cell pool in brain and other tissues. This is caused by chronic decrease in mitochondrial functions and NAD+ levels; nicotinamide phosphoribosyltransferase (Nampt) is the enzyme required to synthesize NAD+. Ablation of Nampt, a rate-limiting enzyme, results in the depletion of neural stem cell pool [143]. Repletion of NAD+ rescues mitochondrial function and increases stem cell pool and thus regenerative capacity by NAD+-induced sirtuin pathways restoring normal mitochondrial functions in aging tissues [144]. In reviewing this body of literature, one must appreciate the differential response of proliferating and quiescent NPCs under oxidative stress conditions.

Overall, these studies indicate the involvement of ROS in the regulation of proliferation and differentiation of NPCs and that the ROS-antioxidant balance is an important factor in this process. As NPCs have the potential for the treatment of different disorders and neurotrauma in the nervous system, it is critical to use translationally related approaches to direct NPCs towards a specific phenotype.

## 12. Conclusion

Redox biology is a fast expanding field in modern biology. The advances in biochemistry have enabled new techniques to examine the changes in proteins that are mediated by a simple oxidation by oxidizing ROS. The conformational change is fast and can be quickly reversed by oxidoreductase proteins using protons from NADPH. This form of signaling is increasingly identified in many systems. The level of ROS level determines the outcome: low levels are acting locally in the vicinity of the ROS source such as the membrane NADPH and are involved in signaling, but high levels initiate a full-scale oxidative damage on cellular macromolecules and cause cell damage. Antioxidants are important players that can determine the cellular response. However, evidence of susceptibility of antioxidant proteins to oxidative damage has been shown in the literature, including our group. It is therefore important to carefully dissect the physiological and pathophysiological levels of ROS when addressing any particular effect of ROS in a biological system.

NPCs are located in a low oxygen niche in SVZ and SGZ, and therefore ROS signaling system can effectively regulate their proliferation and differentiation. Cellular antioxidant systems can potentially modulate the ROS levels and therefore must be examined cautiously when addressing the redox-mediated changes in NPCs. Additionally, in vitro examination of NPCs and their response to ROS manipulation is often performed in normal atmosphere (21%) oxygen pressure which is significantly higher than the normal physiological concentration of oxygen (3–5%) in the brain. Therefore, specific attention to details must be applied in these conditions. Introduction of complementary approaches including redox western blotting in these studies can potentially identify new redox-sensitive proteins or systems and expand our understanding in this field. The employment of powerful genetic modifications has enabled in vivo examination of NPCs; however, using knockout animal models is always associated with potential upregulation of compensatory systems that may obscure the results.

Age-dependent or disease-associated changes in the redox status of the brain suggest that antioxidant therapy may be a potential therapeutic application or intervention to induce NPCs healing capacity. It must be noted that although targeting ROS scavenging has constantly failed to produce any results, the quest for finding novel therapies that can modulate the cellular reducing capacity may be an effective approach to block ROS-mediated effects in CNS. Future therapy shall be formulated in fine-tuning the redox balance utilizing antioxidants and redox-sensitive proteins.
